# Ultra-High-Frequency Ultrasound of Oral Cavity Lesions: A Retrospective Pictorial Study with Histopathologic and Clinical Correlation

**DOI:** 10.3390/diagnostics16172809

**Published:** 2026-09-01

**Authors:** Anna Russo, Vittorio Patanè, Stefano Lucà, Fabrizio Urraro, Nicoletta Giordano, Fabrizio Chirico, Mario Santagata, Marco Montella, Alfonso Reginelli

**Affiliations:** 1 Precision Medicine Department, University of Campania “Luigi Vanvitelli”, 80138 Naples, Italy; 2 Department of Mental and Physical Health and Preventive Medicine, University of Campania “Luigi Vanvitelli”, 80138 Naples, Italy; 3 Department of Life Sciences, Health and Health Professions, Link Campus University, 00165 Rome, Italy; 4 Oral and Maxillofacial Surgery Unit, Multidisciplinary Department of Medical-Surgical and Dental Specialties, University of Campania “Luigi Vanvitelli”, 80138 Naples, Italy

**Keywords:** ultra-high frequency ultrasound, intraoral ultrasound, oral cavity, oral mucosa, oral squamous cell carcinoma, depth of invasion, tumor thickness, histopathology, Doppler ultrasound, mucosal lesions, oral imaging

## Abstract

**Background:** Ultra-high-frequency ultrasound (UHFUS), performed in the present study using 48 and 70 MHz transducers, enables high-resolution assessment of superficial tissues and may provide useful information on the morphology, internal architecture, vascularity, and anatomical relationships of oral cavity lesions. **Methods:** This retrospective single-center study included consecutive patients examined between January 2025 and June 2026. Of 137 eligible cases, five were excluded because of inadequate image or cine-loop quality, leaving 132 lesions for analysis. All examinations were performed by the same experienced radiologist using 48 and 70 MHz linear probes. Each lesion was assessed in B-mode in at least two orthogonal planes and with Color Doppler. Archived anonymized examinations were reviewed using a predefined structured form. Morphology, margins, echogenicity, echotexture, composition, posterior acoustic features, vascularity, dimensions, presumed plane of origin, and involvement of adjacent anatomic layers were evaluated. The qualitative synthesis was performed jointly by a radiologist and an oral pathologist, with disagreements resolved by consensus. **Results:** UHFUS allowed detailed visualization of mucosal, submucosal, muscular, and bone/periodontal interfaces and enabled qualitative characterization of lesion boundaries, internal structure, vascular patterns, and local extension. The combined use of 48 and 70 MHz probes provided complementary information according to lesion depth and tissue composition. Five representative histologically confirmed cases from the study cohort illustrated reactive/inflammatory, benign neoplastic, and malignant conditions. One additional clinically confirmed gingival fistula, not included in the analytical cohort, was presented solely as an illustrative example of a superficial fistulous tract detectable with UHFUS. **Conclusions:** UHFUS provides detailed, layer-based assessment of oral cavity lesions and may complement clinical examination and histopathology in lesion characterization and preoperative evaluation. Sonographic findings should be interpreted within a multimodal diagnostic framework rather than as stand-alone criteria.

## 1. Introduction

Oral cavity lesions represent a heterogeneous group of disorders ranging from reactive and inflammatory changes to benign tumors, salivary gland lesions, potentially malignant disorders, and invasive malignancies [1,2]. Despite this heterogeneity, many lesions share the same initial clinical condition: they are visible or palpable abnormalities of the oral mucosal surface [3,4,5]. This creates a diagnostic challenge because the visible surface is only one part of the disease [6]. The biological and therapeutic relevance of an oral lesion depends on what happens beneath the surface: whether the lesion remains epithelial or mucosal, whether it expands into the submucosa, whether it distorts or infiltrates muscular planes, whether it is associated with altered vascularity, and whether it approaches periodontal or bone-adjacent interfaces [7,8].

From a first-principles perspective, the essential diagnostic questions are spatial and structural before they are nominal. The first question is not simply “what is the lesion called?” but “where is the lesion, which layer does it involve, how far does it extend, what are its boundaries, and how does it interact with the surrounding tissue architecture?” Clinical inspection can identify color, ulceration, surface irregularity, plaque-like change, or exophytic growth [9,10]. Palpation can suggest firmness, tenderness, mobility, or fixation. Histopathology establishes the final diagnosis. However, the interval between clinical examination and histology contains an important information gap: the lack of a real-time, high-resolution map of the lesion within the superficial oral tissues [11,12,13].

Computed tomography and magnetic resonance imaging are fundamental in the staging of advanced oral cavity tumors and in the assessment of deep extension, bone involvement, nodal disease, and complex anatomical spread [14,15,16]. Their role is less direct when the lesion is small, superficial, or confined to the mucosal and submucosal planes. In these settings, clinically important distances may be very small [17,18,19,20]. A few millimeters may separate a superficial benign lesion from a lesion with submucosal extension, and in oral squamous cell carcinoma, depth-related parameters such as tumor thickness and depth of invasion have major prognostic and therapeutic implications [21,22]. Therefore, a method designed to resolve superficial tissue layers may provide information that conventional clinical examination cannot provide and that broader cross-sectional imaging may not optimally capture.

Ultrasound generates images from acoustic interfaces, with frequency determining the fundamental trade-off between spatial resolution and tissue penetration [23,24]. UHFUS exploits very high frequencies to maximize depiction of superficial structures at the expense of penetration depth This trade-off is particularly suited to the oral cavity, where the mucosa, submucosa, minor salivary glands, muscular planes, periodontal structures, and cortical bone interfaces are arranged within relatively superficial tissue compartments. Accordingly, intraoral UHFUS may characterize lesion boundaries, internal echostructure, vascularity, and relationship with site-specific anatomical layers. Because normal sonographic anatomy varies among oral subsites, interpretation requires recognition of the relevant mucosal, submucosal, muscular, glandular, periodontal, and cortical landmarks. This trade-off is not a weakness when the target is shallow. It is the reason the technique is biologically and anatomically suited to superficial tissues.

The oral cavity is a particularly rational target for UHFUS. The mucosa, lamina propria, submucosa, minor salivary glands, adipose lobules, muscle fibers, gingival interfaces, periodontal structures, and cortical bone surfaces are arranged in shallow layers. Many oral lesions arise within these layers. In such a setting, the core strength of UHFUS is not merely lesion detection. In most cases, the lesion has already been detected clinically. The value of UHFUS is lesion characterization: converting a visible mucosal abnormality into a measurable anatomical object with definable boundaries, internal echostructure, vascular behavior, and layer involvement.

This layer-based concept is central. A hypoechoic nodule, an exophytic mucosal lesion, or a heterogeneous submucosal mass cannot be interpreted correctly without knowing the normal architecture of the site in which it occurs. The tongue, buccal mucosa, gingiva, lip, and hard palate differ in their normal sonographic anatomy. In the tongue, the mucosa, submucosa, muscle fibers, and fibrous septum provide landmarks for determining whether a lesion is superficial or deeply infiltrating. In the buccal mucosa, the relationship among mucosa, submucosa, adipose lobules, connective tissue, and buccinator muscle helps distinguish confined mucosal lesions from deeper processes. In the gingiva, the relationship with the mucogingival junction, periodontal region, and alveolar bone interface is essential. In the lip, minor salivary glands and orbicularis oris muscle provide important reference structures. In the hard palate, the fibrous submucosa and cortical bone interface create a strong acoustic boundary that is useful but also technically limiting.

The current literature supports the development of intraoral ultrasound and UHFUS in this direction [25,26,27,28,29]. Anatomical studies have shown that UHFUS can depict normal oral mucosa and characterize site-specific biomarkers such as epithelial thickness, echogenicity, and vascularization [30]. Other investigations have focused on intraoral ultrasound for oral squamous cell carcinoma, especially preoperative assessment of tumor thickness and depth of invasion, with histopathology as the reference standard [31,32]. Systematic reviews and recent prospective studies suggest that ultrasound can correlate with histological depth-related measurements and may compare favorably with magnetic resonance imaging in selected early tongue cancers [33]. At the same time, the evidence remains heterogeneous, with variation in probes, frequencies, acquisition protocols, measurement definitions, lesion sites, and study endpoints [34,35]. This heterogeneity limits immediate standardization and highlights the need for structured, site-aware, histologically correlated clinical experiences.

Against this background, the originality of the present study is its layer-based framework. Rather than focusing exclusively on oral squamous cell carcinoma or on a single anatomical site, this work applies intraoral UHFUS to a broader spectrum of oral cavity lesions and interprets each lesion through the same anatomical and morphological logic: site, layer of origin, margins, echogenicity, echostructure, vascularity, and depth-related extension. Histopathology remains the reference standard, but UHFUS is evaluated as a bridge between clinical examination and tissue diagnosis.

The primary aim of this retrospective single-center observational study was to evaluate intraoral UHFUS for the characterization of oral cavity lesions using histopathology as the reference standard. Secondary aims were to describe the normal UHFUS anatomy of selected oral subsites, assess the feasibility and limitations of intraoral scanning, describe the sonographic features observed across histopathologically confirmed benign/reactive and malignant lesions, and place our findings within the context of the current literature on oral mucosal imaging, tumor thickness, and depth of invasion.

## 2. Methods

The study was conducted in accordance with the Declaration of Helsinki and approved by the Ethics Committee of the University Hospital of Campania “L. Vanvitelli” (protocol code 59/2017; approval date: 10 November 2017). The requirement for informed consent for participation was waived by the Ethics Committee because of the retrospective nature of the study and the use of anonymized data. Written informed consent for the publication of anonymized clinical and imaging material was obtained from the patients represented in the figures.

### 2.1. Study Design

This was a retrospective, single-center observational original study designed to evaluate the role of intraoral UHFUS in patients with clinically detectable oral cavity lesions. The study included a pictorial component in which selected representative cases were used to illustrate the principal imaging–pathology correlations observed within the cohort; however, these cases were derived from the original retrospective study population and did not constitute an independent case series or narrative review. UHFUS was considered the index imaging test, while histopathological examination after biopsy or surgical excision was considered the reference standard.

The study was not designed as a screening study. Its purpose was not to determine whether UHFUS can replace clinical examination, histopathology, computed tomography, or magnetic resonance imaging. Instead, the study was based on a more focused diagnostic question: can intraoral UHFUS provide structured, clinically meaningful, layer-based information about superficial oral lesions before histological confirmation?

The core mechanism underlying this approach is anatomical matching. UHFUS is most useful when the imaging scale matches the disease scale. Oral mucosal and submucosal lesions often occupy superficial tissue compartments; UHFUS provides high-resolution information within these superficial compartments. Therefore, the study evaluated whether the technique could define lesion morphology, internal structure, vascularity, and relationship with adjacent layers.

### 2.2. Study Population

The final study population included 132 patients evaluated between January 2025 and June 2026 at University Hospital “Luigi Vanvitelli” in Naples. Patients were eligible when they presented with a clinically detectable oral cavity lesion and underwent intraoral UHFUS before biopsy or surgical excision.

Inclusion criteria were: (1) presence of a visible or palpable lesion of the oral cavity; (2) lesion site accessible to intraoral UHFUS scanning; (3) availability of histopathological diagnosis; (4) UHFUS performed before definitive treatment or histopathological interpretation; and (5) complete clinical and imaging documentation.

Exclusion criteria were: (1) inability to tolerate intraoral probe positioning; (2) inaccessible lesion site because of posterior location, severe gag reflex, limited mouth opening, pain, or anatomical constraints; (3) inadequate image quality due to motion, acoustic contact failure, or artifacts; (4) absence of histopathological confirmation; and (5) incomplete data preventing imaging–pathology comparison.

Demographic and clinical data included age, sex, lesion site, clinical indication, symptoms when available, and final histopathological diagnosis. The analytical cohort comprised exclusively histopathologically confirmed lesions. Five illustrative cases presented in the figures were selected from this cohort for educational purposes. One additional gingival fistula without histopathological sampling was included solely as an illustrative clinical example and was not considered part of the 132-lesion analytical cohort or included in any cohort-level analysis.

### 2.3. Ultrasound Equipment

All examinations were performed using a Fujifilm Vevo MD (FUJIFILM VisualSonics, Toronto, ON, Canada) ultra-high-frequency ultrasound system equipped with linear UHF48 and UHF70 transducers. Both transducers were used as components of the same UHFUS examination rather than as alternative or competing tests. The UHF48 transducer provides greater imaging depth, approximately 23 mm, with a nominal image resolution of approximately 50 μm, whereas the UHF70 transducer provides higher superficial resolution, approximately 30 μm, with an imaging depth of approximately 10 mm. The two probes were therefore used in a complementary manner to exploit the inherent trade-off between spatial resolution and acoustic penetration. The operator integrated the information obtained with both frequencies according to lesion morphology, accessibility, and expected depth. Greater emphasis was placed on the 70 MHz images for very superficial structures and fine anatomical interfaces, whereas the 48 MHz images were particularly useful when a greater depth of interrogation was required. No predefined probe-specific diagnostic threshold was used, and the study was not designed to determine the incremental diagnostic value or superiority of either transducer. No fixed sequence of probe application was mandated; the order was determined by the operator according to the lesion characteristics and anatomical accessibility. This individualized frequency selection reflects the intrinsic physical trade-off of UHFUS: increasing frequency improves the depiction of fine superficial structures but increases acoustic attenuation and consequently reduces tissue penetration. Conversely, the relatively lower 48 MHz frequency provides greater penetration at the expense of some spatial detail.

This limitation was considered acceptable for the present application because many oral cavity lesions are located within the mucosal or submucosal layers. When a lesion was suspected to extend beyond the field of view or to involve deep structures not adequately assessable with UHFUS, cross-sectional imaging remained necessary according to clinical judgment.

### 2.4. Scanning Protocol

The patient was examined with the mouth partially open. This position was used to reduce excessive soft-tissue traction and facilitate stable intraoral probe placement. All UHFUS examinations were performed and interpreted by a single operator with 15 years of experience in dermatologic and soft-tissue ultrasonography. Image interpretation was performed before histopathological results were available and therefore independently of the final histopathological diagnosis. The transducer was covered with a sterile sheath. Acoustic coupling was obtained with gel placed inside the cover or with chlorhexidine according to local practice.

The probe was gently placed in contact with the mucosal surface overlying or adjacent to the lesion. Excessive compression was avoided because superficial tissues are deformable, and pressure may alter lesion thickness, distort the relationship among layers, and reduce Doppler signal. The operator acquired axial and sagittal B-mode scans in all cases. Color Doppler imaging was systematically performed in all 132 included examinations to assess lesion vascularity and its distribution. Particular care was taken to avoid excessive transducer pressure, as compression of superficial tissues may reduce or suppress low-flow vascular signals. Although Doppler acquisition may be technically more demanding in posterior or poorly accessible locations because of limited probe maneuverability, motion, patient discomfort, gag reflex, or difficulty in maintaining stable contact, these factors did not prevent Color Doppler assessment in the included examinations.

The scanning approach was adapted to the lesion morphology and anatomical site. For exophytic lesions, the base of implantation, superficial extension, and relationship with mucosa and submucosa were assessed. For submucosal lesions, the examination focused on depth, margins, echostructure, vascularity, and displacement or infiltration of adjacent layers. For gingival, periodontal, or hard-palate lesions, attention was given to the relationship with the alveolar or palatal cortical interface, recognizing that bone produces posterior acoustic shadowing and limits assessment of structures beyond the cortex.

### 2.5. Normal Anatomical Reference Model

Image interpretation followed a site-specific anatomical model. This was necessary because UHFUS findings are meaningful only when interpreted against the expected normal architecture of each oral subsite.

In the tongue, the mucosa was expected to appear as a superficial hypoechoic layer. The submucosa appeared as a deeper hyperechoic layer. The muscular plane appeared thicker and heterogeneous, with intrinsic and extrinsic muscle fibers producing internal echogenic lines. The fibrous septum, when visible, appeared as a median linear hyperechoic structure.

In the buccal mucosa, the superficial mucosa appeared thin and hypoechoic. The underlying submucosa appeared thicker and hyperechoic. Hypoechoic lobular structures within the submucosa were interpreted as adipose tissue surrounded by connective tissue. The buccinator muscle appeared as a deeper heterogeneous hypoechoic structure with internal hyperechoic fibrils.

In the gingiva, the assessment included the superficial mucosa, submucosa, free or marginal gingiva, gingival sulcus, attached gingiva, mucogingival junction, periodontal region, and alveolar bone interface. The alveolar cortical boundary appeared as a strong hyperechoic line with posterior shadowing.

In the lip, the mucosa appeared as a superficial hypoechoic layer, while the submucosa appeared hyperechoic because of collagenous and elastic components. Minor salivary glands could be recognized as round, homogeneous, hypoechoic structures within the submucosa. The orbicularis oris muscle appeared as a deeper hypoechoic muscular layer with internal fibrillar echoes.

In the hard palate, the mucosa appeared as a narrow superficial hypoechoic layer, the fibrous submucosa as a hyperechoic layer, and the palatal cortical bone as a markedly hyperechoic band with posterior acoustic shadowing. This site was considered technically challenging because of rigidity, curvature, limited probe maneuverability, and strong acoustic reflection from bone.

### 2.6. Imaging Variables

Each lesion was evaluated using predefined variables. Anatomical site was recorded as tongue, buccal mucosa, gingiva, lip, hard palate, sublingual region, or other oral cavity site. Morphology was described as round, oval, exophytic, nodular, plaque-like, polylobulated, or infiltrative. Margins were described as well-defined, regular, irregular, stellate, or poorly defined.

Maximum diameter was measured along the predominant axis. Additional orthogonal diameters were recorded when available. Echogenicity was classified relative to surrounding tissue as hypoechoic, isoechoic, hyperechoic, or mixed. Echostructure was classified as homogeneous or heterogeneous. Doppler vascularity was categorized as absent, mild, moderate, or marked, and its distribution was recorded as peripheral, central, or mixed.

Layer involvement was defined according to the relationship between the lesion and the normal anatomical planes. A lesion was considered mucosal when confined to the superficial mucosal layer. It was considered submucosal when it extended into or arose from the submucosa. Muscular involvement was suspected when the lesion interrupted, distorted, or infiltrated the muscular plane. Bone-adjacent involvement was considered when the lesion reached the cortical interface or altered the expected relationship with bone. UHFUS was not considered sufficient to exclude deep bone invasion when clinical suspicion required computed tomography or magnetic resonance imaging.

### 2.7. Histopathological Reference Standard

Histopathological examination was used as the reference standard for the final diagnosis of all included lesions. Biopsy or surgical excision specimens were processed according to standard pathological practice, and lesions were categorized according to the final histopathological diagnosis. When available, pathological information regarding tumor thickness, depth of invasion, layer involvement, tissue type, and lesion dimensions was recorded for descriptive imaging–pathology correlation. However, UHFUS and histopathological measurements were not prospectively co-registered along a predefined identical anatomical axis, and no correction for tissue deformation or shrinkage related to fixation and histological processing was applied. Consequently, histopathological measurements were not used for formal quantitative agreement analysis with UHFUS measurements. Tumor thickness and depth of invasion were treated as distinct pathological parameters and were not used interchangeably.

### 2.8. Statistical Analysis

Because this was a retrospective study with primarily descriptive and qualitative objectives, statistical analyses were designed to summarize the characteristics of the study population rather than to test a prespecified inferential hypothesis. The study population consisted of all consecutive patients fulfilling the predefined eligibility criteria during the study period; therefore, no a priori sample-size calculation was performed.

Continuous variables were summarized descriptively using measures of central tendency and dispersion, as appropriate to their distribution, whereas categorical variables were reported as absolute numbers and percentages.

No formal between-group hypothesis testing was performed, as the study was not designed to establish statistically validated differences between benign and malignant lesions or between anatomical subsites. Similarly, no sensitivity, specificity, predictive values, or other diagnostic performance metrics were calculated because no prespecified imaging threshold or binary diagnostic classification rule had been defined.

Formal agreement analysis between UHFUS and histopathological quantitative measurements was not performed in the present study. In addition, because all examinations and the retrospective image review were performed by a single radiologist, no independent second-reader dataset was available for inter-observer agreement analysis, and no dedicated repeatability assessment was performed for intra-observer reliability.

Given the absence of a prespecified inferential primary endpoint or expected effect size, no post hoc power analysis was performed. The statistical results should therefore be interpreted as descriptive and exploratory.

## 3. Results

### 3.1. Patient and Lesion Characteristics

A total of 132 patients were included in the final analysis, corresponding to 132 oral cavity lesions. The cohort consisted of 47 women (35.6%) and 85 men (64.4%), with a mean age of 65.2 ± 12.7 years. Lesions were located in the tongue (*n* = 25, 18.9%), buccal mucosa (*n* = 22, 16.7%), gingiva (*n* = 13, 9.8%), lip (*n* = 7, 5.3%), hard palate (*n* = 8, 6.1%), sublingual region (*n* = 15, 11.4%), and other oral cavity sites (*n* = 42, 31.8%). Histopathological examination demonstrated 57 benign/reactive lesions (43.2%) and 75 malignant lesions (56.8%). The demographic, anatomical, and histopathological characteristics of the study cohort are summarized in Table 1.

### 3.2. Feasibility and Technical Performance

Intraoral UHFUS yielded technically adequate and interpretable examinations in 132 of the 137 patients in whom imaging was performed, corresponding to an observed technical feasibility of 96.4%. Five examinations (3.6%) were excluded because image and/or cine-loop quality was insufficient for reliable retrospective interpretation. Among technically successful examinations, the procedure was generally rapid and well tolerated, particularly for anterior and easily accessible lesions. The examination was generally rapid and well tolerated, particularly for anterior and easily accessible lesions. Image quality was best when the probe could be placed in stable contact with the mucosal surface and the lesion could be scanned in both axial and sagittal planes.

Technical limitations were primarily related to anatomical access. Posterior and lateral locations were more difficult because of limited space, patient discomfort, gag reflex, and reduced maneuverability of the probe. The hard palate was also technically challenging because of its curved rigid surface and the strong cortical bone reflection that produced posterior acoustic shadowing. These findings define the practical field of UHFUS: it is most suitable for superficial, accessible lesions and less suitable as a standalone method for deeply located, posterior, or extensive disease.

### 3.3. Normal UHFUS Anatomy of the Oral Cavity

UHFUS provided a consistent depiction of oral cavity layers. Across subsites, the mucosa was generally visible as a superficial hypoechoic band. The submucosa appeared as a deeper hyperechoic layer, although its appearance varied according to the proportion of collagen, elastic fibers, adipose tissue, glands, and connective tissue. Muscular structures appeared as thicker hypoechoic or heterogeneous layers with internal hyperechoic fibrils. Cortical bone appeared as a highly reflective hyperechoic interface with posterior acoustic shadowing.

In the tongue, UHFUS allowed recognition of the superficial mucosa, submucosa, and muscular architecture. The ability to distinguish these layers was central for determining whether a lesion was superficial or whether it extended toward deeper muscular planes.

In the buccal mucosa, UHFUS distinguished the mucosal and submucosal layers and showed deeper muscular landmarks corresponding to the buccinator muscle. Hypoechoic lobular structures within the submucosa were interpreted as adipose components within connective tissue. This anatomical reference was useful for evaluating whether a lesion was confined to the mucosal surface, located within the submucosa, or approaching the muscular plane.

In the gingiva, UHFUS depicted the mucosal and submucosal components and the relationship with the alveolar bone interface. The gingival margin, attached gingiva, mucogingival junction, and periodontal region could be evaluated when technically visible. The strong cortical reflection from alveolar bone helped define the deep boundary of the superficial gingival tissues.

In the lip, UHFUS identified the mucosa, submucosa, minor salivary gland structures, and orbicularis oris muscle. This allowed differentiation between exophytic mucosal lesions and deeper submucosal or muscular processes.

In the hard palate, UHFUS showed the superficial mucosa, hyperechoic fibrous submucosa, and the palatal cortical bone interface. This anatomical pattern was useful for identifying superficial lesions but also demonstrated the physical limitation created by bone shadowing.

### 3.4. UHFUS Features of Benign Lesions

Within the benign/reactive group, the sonographic appearances included well-defined margins, localized morphology, preservation of deeper anatomical planes, and, in some exophytic lesions, confinement to the mucosal layer or a clearly defined base without evidence of deep infiltration. Some submucosal benign lesions showed displacement rather than overt disruption of surrounding tissues. These observations are descriptive and were not subjected to formal frequency-based or between-group statistical comparison; histopathological confirmation therefore remained necessary. A post-traumatic lingual fibroma appeared as an exophytic isoechoic lesion with well-defined margins, located above the mucosal region without submucosal extension. This finding illustrates a key practical function of UHFUS: confirming whether a clinically superficial lesion is actually limited to superficial layers.

**Figure 1:**
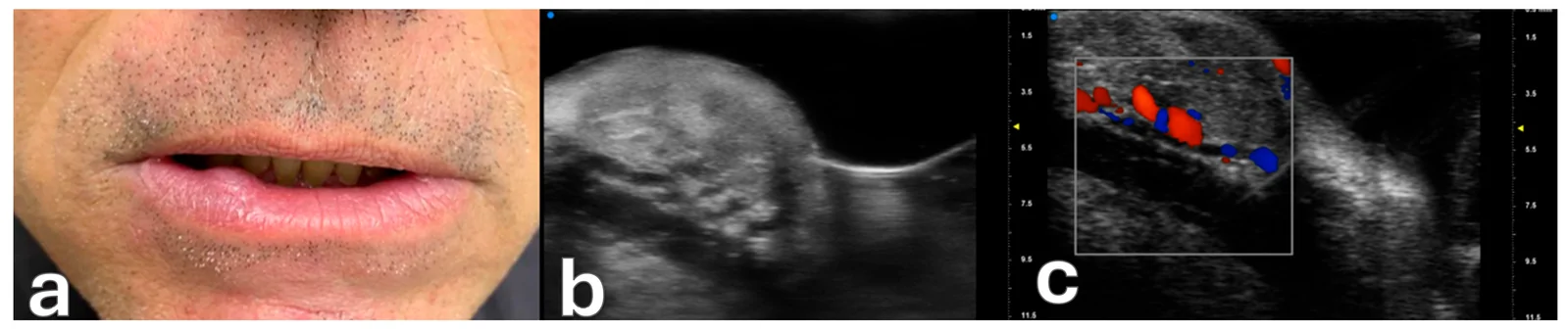
Benign vascular lesion of the lower lip evaluated with intraoral ultra-high frequency ultrasound. (**a**) Clinical photograph showing a subtle exophytic lesion of the lower lip mucosa. (**b**) B-mode UHFUS demonstrates a well-defined, predominantly hyperechoic mucosal lesion with regular margins and no evident infiltration of the deeper planes. (**c**) Color Doppler image shows moderate vascular signal, mainly distributed at the periphery of the lesion. The combined clinical and sonographic pattern was consistent with a benign vascular lesion; histopathological correlation suggested angioleiomyoma.

A benign lower-lip lesion, considered compatible with angioleiomyoma, appeared as a well-defined exophytic hyperechoic mucosal lesion with moderate predominantly peripheral vascularization (Figure 1). In this case, Doppler evaluation provided additional functional information by showing the distribution of flow around the lesion.

**Figure 2:**
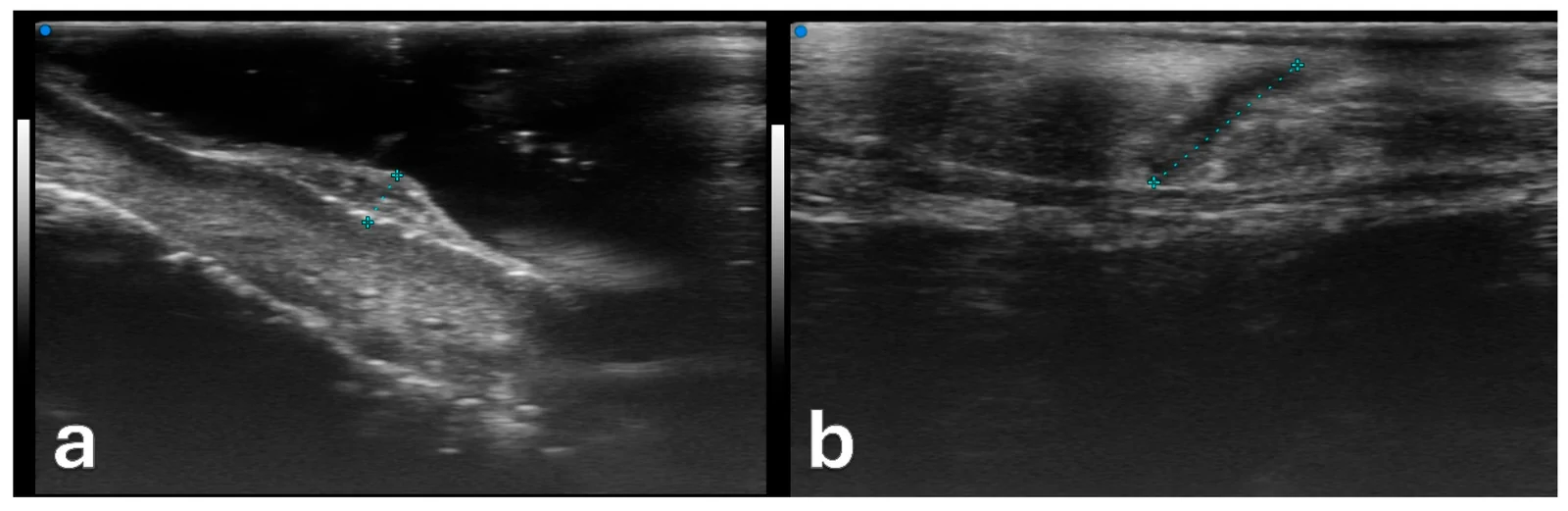
Additional clinically confirmed gingival fistula outside the histologically confirmed study cohort. (**a**) Long-axis UHFUS image shows a small exophytic lesion arising from the upper right gingival region, with an internal echogenic component consistent with keratinic content. (**b**) A second UHFUS image demonstrates an underlying hypoechoic tract extending beneath the lesion, interpreted as a fistulous pathway communicating with the exophytic gingival lesion. No histopathological sampling was performed in this case; the diagnosis was supported by maxillofacial surgical assessment and subsequent clinical resolution. This patient was not included in the 132-lesion analytical cohort.

As an additional illustrative example outside the histologically confirmed study cohort, a gingival lesion in a young adult showed an exophytic granulomatous component with internal keratinic content and an underlying hypoechoic tract interpreted as a fistulous pathway communicating with the lesion (Figure 2). This case was not included in the 132-lesion analytical cohort because no histopathological sampling was performed. The clinical diagnosis was supported by maxillofacial surgical assessment and subsequent clinical resolution.

This case suggests that UHFUS may be useful not only for mass characterization but also for identifying superficial tracts, fistulous connections, or inflammatory extensions.

**Figure 3:**
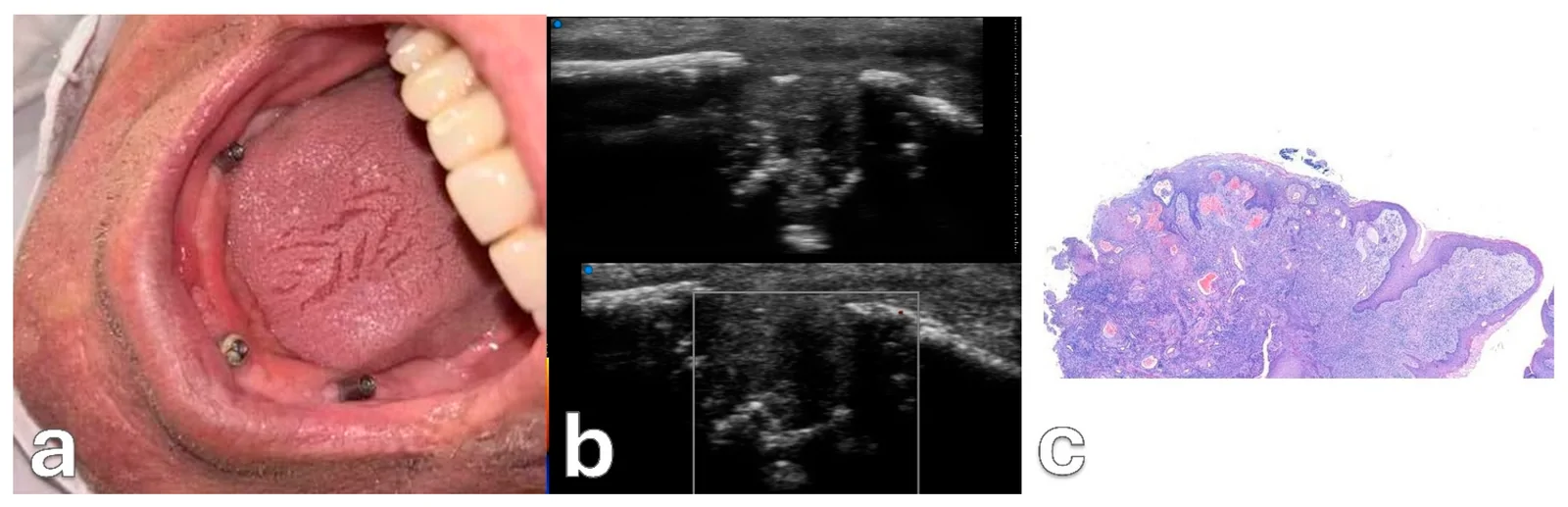
Pyogenic granuloma of the mandibular alveolar crest after implantology evaluated with intraoral ultra-high frequency ultrasound and histopathological correlation. (**a**) Clinical photograph showing a small mucosal lesion in the right mandibular alveolar crest region, adjacent to dental implants. (**b**) B-mode UHFUS performed with a 48 MHz probe demonstrates an irregular cortical profile, consistent with post-surgical changes, and a rounded iso-hyperechoic lesion measuring approximately 4.90 × 6.04 × 4.48 mm. The lesion is located superficially at the mucosal/peri-implant interface. (**c**) Histopathological specimen confirming pyogenic granuloma. This case illustrates the ability of UHFUS to characterize reactive peri-implant mucosal lesions and to assess their relationship with the superficial soft tissues and underlying cortical interface.

A hard-palate lesion showed polylobulated morphology, regular margins, finely hypoechoic echostructure, and no significant Doppler signal. In another reactive lesion involving the right mandibular alveolar crest after implantology, UHFUS demonstrated a rounded iso-hyperechoic formation adjacent to an irregular cortical profile, consistent with a post-surgical peri-implant inflammatory lesion. Histopathology confirmed pyogenic granuloma (Figure 3).

**Figure 4:**
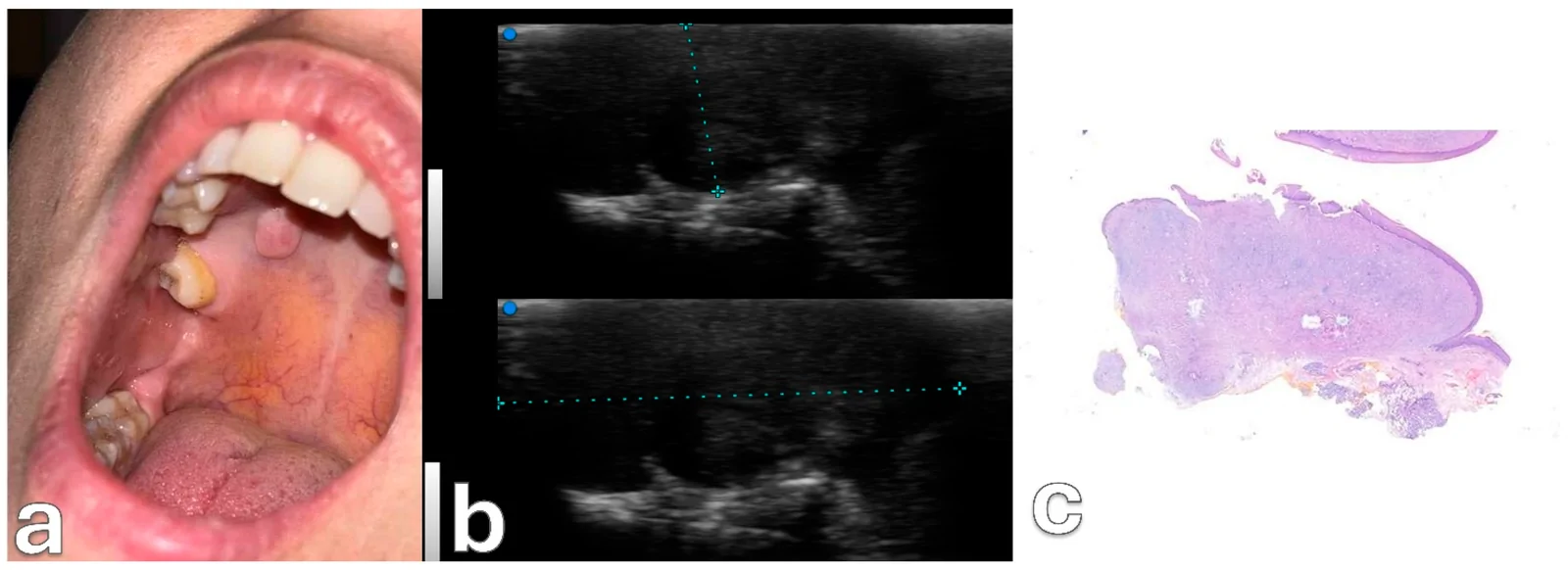
Storiform collagenoma of the hard palate evaluated with intraoral ultra-high frequency ultrasound and histopathological correlation. (**a**) Clinical photograph showing a small polylobulated lesion located in the posterior hard palate. (**b**) B-mode UHFUS performed with a 48 MHz probe demonstrates a superficial polylobulated lesion with regular margins and finely hypoechoic echostructure, measuring approximately 3.76 × 6.58 × 8.97 mm. The lesion is located close to the palatal cortical interface, which appears as a deep hyperechoic boundary with posterior acoustic shadowing. (**c**) Histopathological specimen confirming storiform collagenoma, also known as sclerotic fibroma. The case illustrates the usefulness of UHFUS for characterizing benign hard-palate lesions while also highlighting the technical limitations related to the rigid palatal surface and underlying bone interface.

A hard-palate lesion showed polylobulated morphology, regular margins, finely hypoechoic echostructure, and no significant Doppler signal, measuring approximately 3.76 × 6.58 × 8.97 mm on UHFUS (Figure 4). Histopathology demonstrated storiform collagenoma, also known as sclerotic fibroma.

The sonographic pattern was consistent with a benign lesion because of its regular profile, limited size, and absence of aggressive disruption of adjacent layers.

### 3.5. UHFUS Features of Malignant Lesions

Within the malignant group, observed sonographic findings included irregular or stellate margins, heterogeneous echostructure, hypoechoic components, increased vascularity, and mucosal or submucosal involvement. These findings were not uniformly present and were not quantitatively compared with those observed in benign/reactive lesions. These features are not arbitrary image descriptors; they are imaging consequences of underlying biological mechanisms. Invasive growth disrupts normal boundaries. Tumor cellularity, keratinization, necrosis, fibrosis, and stromal reaction alter echostructure. Neoangiogenesis and inflammatory vascular remodeling may increase the Doppler signal. Therefore, UHFUS signs should be interpreted as macroscopic reflections of tissue organization rather than as isolated visual patterns.

**Figure 5:**
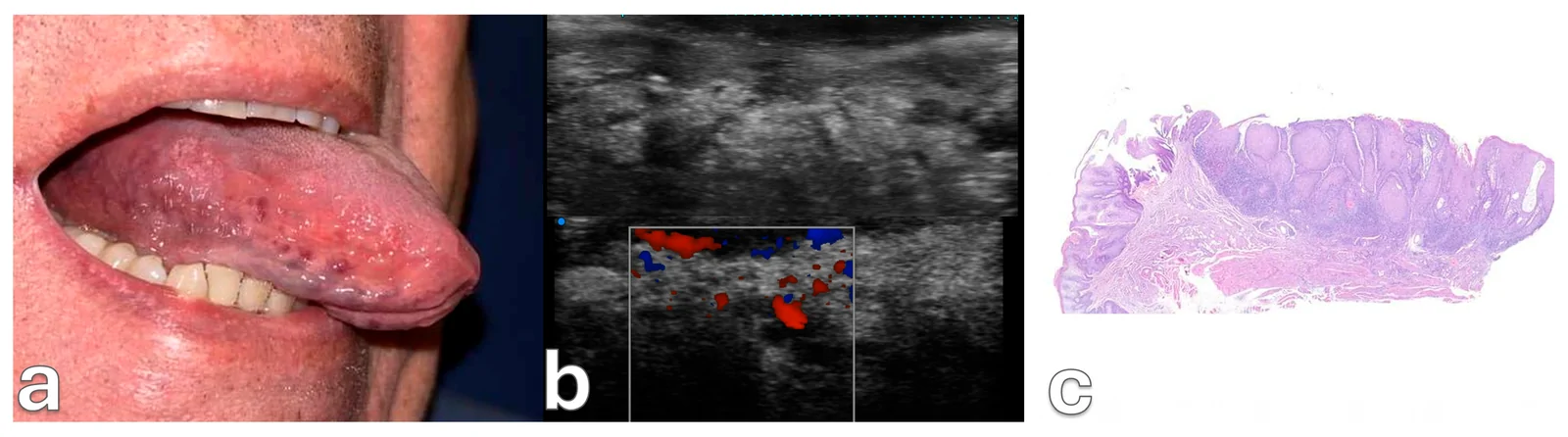
Early squamous cell carcinoma of the lateral tongue evaluated with intraoral ultra-high frequency ultrasound and histopathological correlation. (**a**) Clinical photograph showing a mucosal lesion of the lateral border of the tongue. (**b**) Color Doppler UHFUS demonstrates vascular signal within and around the lesion, with predominantly peripheral vascular distribution. (**c**) B-mode UHFUS shows a superficial lesion with predominantly hypoechoic and mildly heterogeneous echostructure, allowing assessment of its relationship with the mucosal and submucosal planes. Histopathological specimen confirming initially infiltrating grade 1 squamous cell carcinoma. The case illustrates the role of UHFUS in preoperative characterization of superficial oral squamous cell carcinoma, particularly for evaluating lesion morphology, vascularity, and depth-related extension.

One lesion of the lateral right tongue was examined with a 48 MHz probe and appeared as an oval lesion with predominantly hypoechoic echostructure and intense peripheral Doppler vascularization, measuring approximately 14.21 × 5.06 × 2.01 mm (Figure 5).

The lesion showed predominantly mucosal extension without significant infiltration of deeper planes. Histopathology demonstrated initially infiltrating grade 1 squamous cell carcinoma. A malignant lesion of the posterior right buccal mucosa showed heterogeneous echostructure, partly hypoechoic components, irregular stellate margins, and increased vascularization involving the mucosal and submucosal region. These features were considered compatible with oral squamous cell carcinoma and were subsequently correlated with histopathological diagnosis.

**Figure 6:**
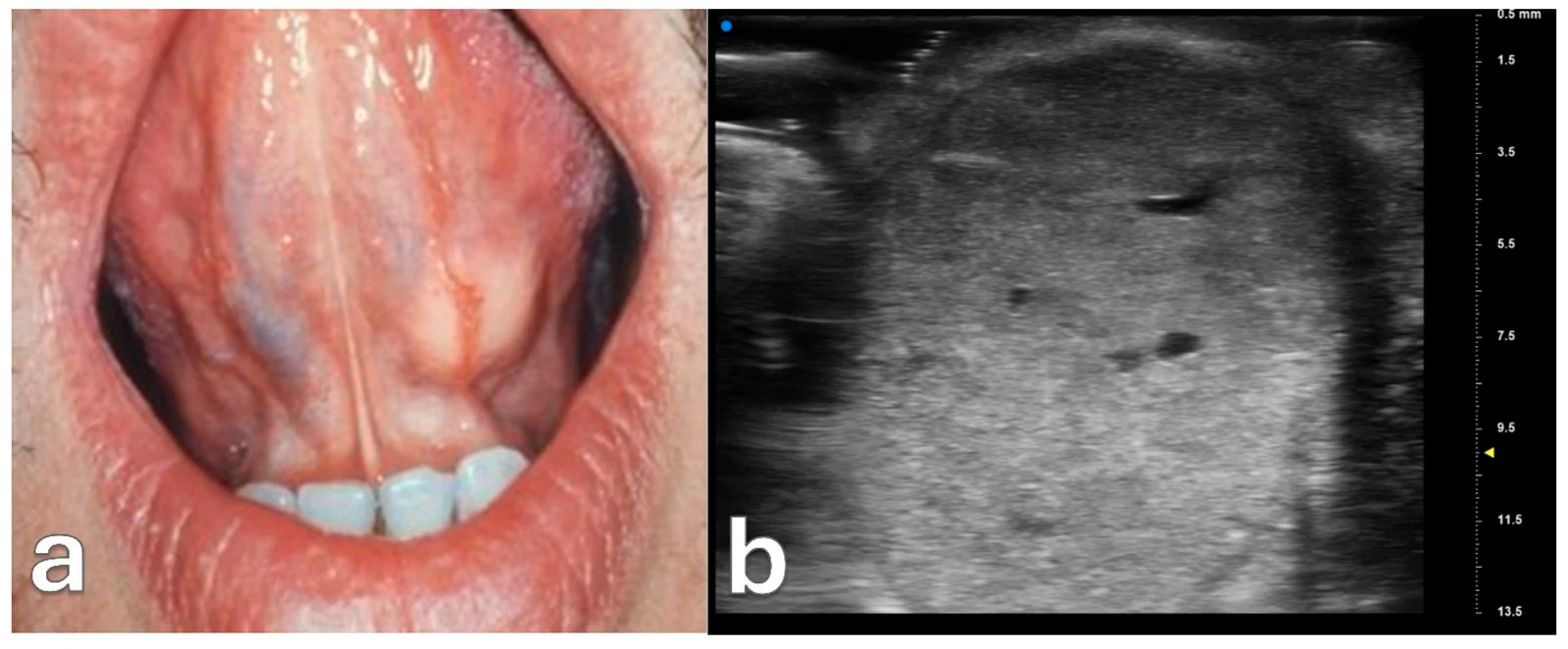
Mucoepidermoid carcinoma of the sublingual gland region evaluated with intraoral ultra-high frequency ultrasound. (**a**) Clinical photograph showing a submucosal swelling in the floor of the mouth/sublingual region. (**b**) B-mode UHFUS demonstrates a rounded submucosal lesion with relatively well-defined margins, increased echogenicity, and finely heterogeneous echostructure. The case emphasizes that malignant salivary gland lesions may show a circumscribed sonographic appearance and that UHFUS should be interpreted as an adjunctive characterization tool rather than a substitute for histopathological diagnosis.

A submucosal lesion of the sublingual gland region in a middle age man was diagnosed histologically as mucoepidermoid carcinoma. On UHFUS, it appeared as a rounded submucosal lesion with well-defined margins, increased echogenicity, and finely heterogeneous echostructure. This case is important because it prevents an overly simplistic interpretation of UHFUS patterns. Not all malignant lesions appear frankly infiltrative (Figure 6).

Some malignant salivary gland tumors may show relatively circumscribed margins, especially when small or confined within a submucosal compartment. Therefore, UHFUS can support characterization, but histopathology remains indispensable.

### 3.6. Histopathological Correlation and Planned Quantitative Outcomes

Histopathological diagnosis was available as the reference standard for all lesions included in the study cohort. Imaging findings were qualitatively interpreted in relation to the corresponding pathological diagnosis, with particular attention to lesion architecture, margins, echotexture, vascularity, presumed plane of origin, and relationship with adjacent tissue layers. The representative cases reported above illustrate the principal imaging–pathology relationships observed in reactive/inflammatory, benign neoplastic, and malignant lesions. No formal quantitative agreement analysis between UHFUS measurements and histopathological measurements was performed.

## 4. Discussion

This retrospective single-center study provides a systematic qualitative account of high- and ultra-high-frequency ultrasound findings in 132 consecutive, histologically confirmed oral cavity lesions. The principal contribution is not the derivation of diagnostic thresholds or estimates of diagnostic accuracy, but the demonstration that a structured, layer-based assessment can be applied across a heterogeneous spectrum of clinically visible or palpable lesions. The combined use of 48 MHz and 70 MHz probes allowed the operator to balance penetration and spatial resolution according to lesion depth and morphology, while B-mode and Color Doppler jointly provided information on lesion architecture, tissue–plane relationships, and vascular pattern. Five histologically confirmed cases from the reviewed cohort and one additional clinically confirmed gingival fistula were selected as illustrative examples of the principal reactive/inflammatory, benign neoplastic, and malignant patterns encountered.

The layer-based approach adopted in this study is supported by previous work on normal oral anatomy. Izzetti et al. showed that intraoral UHFUS can depict site-specific mucosal, submucosal, muscular, glandular, vascular, and cortical interfaces in healthy volunteers, thereby establishing an anatomical benchmark for pathological interpretation [26,30,36]. More recent work focused on the gingiva confirmed that very-high-frequency imaging can delineate the superficial gingival layers and microvascular signals, while also emphasizing the technical difficulty of posterior and curved oral surfaces [17,37,38]. These observations are consistent with our experience: the diagnostic value of a hypoechoic, hyperechoic, or heterogeneous area depends less on its isolated gray-scale appearance than on the layer in which it originates, the planes it displaces or interrupts, and its relationship with muscle, salivary tissue, periodontal structures, or cortical bone.

The closest previous broad clinical investigation is the study by Izzetti et al., which evaluated 160 patients with autoimmune disorders, mucosal growths, potentially malignant lesions, and oral cancer using intraoral UHFUS and histopathological correlation [25]. That study reported reproducible patterns across lesion categories and high diagnostic performance estimates. Our findings support the central premise that superficial oral lesions can be organized according to margins, echogenicity, echostructure, vascularity, and preservation or disruption of tissue layering.

The reactive and benign cases illustrate how UHFUS can add anatomical information to clinical inspection. The peri-implant pyogenic granuloma was characterized not only as a superficial mucosal lesion but also in relation to an irregular postoperative cortical interface. This application is conceptually consistent with the previously reported combined use of UHFUS and cone-beam CT in peri-implant disease, in which ultrasound was used for superficial soft-tissue assessment and CBCT for the osseous component [26]. The present case reinforces the complementary nature of these modalities: UHFUS can characterize the accessible soft-tissue component and its vascular behavior, whereas cross-sectional or dental imaging remains necessary when the clinically relevant question concerns deeper bone or implant-related complications.

The gingival fistula further illustrates the ability of UHFUS to display non-mass-forming disease. The hypoechoic tubular tract, its mildly hyperechoic wall, and heterogeneous surrounding tissue provided an anatomical correlate for the clinical suspicion of a superficial inflammatory pathway. This observation is concordant with a recent gingival UHFUS study in which an otherwise clinically subtle fistulous tract was identified during high-resolution examination [17]. In our case, the absence of histopathological sampling requires explicit distinction from the histologically confirmed cohort; nevertheless, the diagnosis was supported by maxillofacial surgical assessment and by clinical resolution approximately 40 days after antiseptic oral care and smoking cessation. The case therefore demonstrates a potential role for UHFUS in mapping superficial tracts and inflammatory extensions, while also showing that imaging–pathology correlation is not always the most appropriate reference pathway for lesions managed conservatively.

The angioleiomyoma and storiform collagenoma cases highlight a different limitation of pattern recognition. Oral angioleiomyoma is uncommon, and published imaging reports describe a circumscribed soft-tissue mass with Doppler-detectable vascularity, but the final diagnosis remains histological because similar appearances may occur in other vascular or smooth-muscle lesions [39]. Likewise, storiform collagenoma is a rare, usually well-circumscribed fibrous lesion of the oral mucosa [40]. Its regular contour and limited disruption of adjacent layers were compatible with a benign process, but they were not histotype-specific. These cases support a clinically useful distinction: UHFUS may define whether a lesion is circumscribed, vascular, solid, superficial, or bone-adjacent, but it cannot directly identify the cellular and stromal criteria required for a definitive pathological diagnosis.

The malignant examples similarly show both the value and the limits of sonographic morphology. The lateral-tongue squamous cell carcinoma appeared predominantly hypoechoic and mildly heterogeneous, with vascular signal and definable superficial extension. Earlier studies of intraoral ultrasound in tongue masses demonstrated that lesions can be assessed in relation to the intrinsic muscular architecture and that their internal echogenicity and boundary characteristics may support differential characterization [41]. Doppler studies have also linked vascular features at the invasion front of tongue carcinoma with pathological aggressiveness and nodal risk [42]. In the present study, however, Color Doppler was interpreted qualitatively; consequently, vascularity should be regarded as one component of an integrated morphological assessment and not as an independent marker of malignant behavior.

A substantial proportion of the intraoral ultrasound literature has concentrated on tumor thickness and depth of invasion in oral squamous cell carcinoma. A preliminary UHFUS experience from our institution reported strong agreement between ultrasonographic and histopathological measurements of tumor thickness and depth of invasion [19,43]. Systematic reviews and meta-analyses have generally supported a close relationship between intraoral ultrasound and histopathological measurements, while also identifying heterogeneity in probe frequency, measurement definitions, anatomical site, and study design [31,44]. The most recent meta-analysis with trial sequential analysis concluded that ultrasound measurements are promising, particularly for tumor thickness, but that the available evidence remains insufficient to establish universal clinical implementation for depth of invasion [45]. These findings justify a cautious position: high-resolution intraoral ultrasound has clear potential for depth-related assessment, but standardized acquisition, anatomical landmarks, and outcome definitions remain prerequisites for generalizable thresholds.

Prospective and surgical studies provide additional evidence for the role of ultrasound in selected oral tongue cancers. Caprioli et al. reported high correlation and close agreement between intraoral ultrasound and pathological depth of invasion in early tongue squamous cell carcinoma [10]. In a prospective comparison, Kaltoft et al. found smaller measurement differences and more accurate T-stage classification with intraoral ultrasound than with MRI in a cohort dominated by T1-T2 tongue tumors [33]. Bulbul et al. also described the use of intraoperative ultrasound to support deep-margin clearance during oral tongue resection [46]. These studies address quantitative oncological endpoints that were not tested in the present work. Nevertheless, they support the practical concept illustrated by our cases: real-time delineation of the deep lesion boundary may assist procedural planning when the lesion is superficial and accessible. Such use should not be extrapolated to deeply infiltrative, posterior, or extensive disease, for which MRI and CT remain indispensable.

The mucoepidermoid carcinoma is particularly instructive because it appeared relatively rounded and well circumscribed. This finding cautions against equating regular margins with benignity. Small salivary-type malignancies may expand within a submucosal compartment before producing overtly infiltrative imaging features. Conversely, inflammatory and reactive lesions may be heterogeneous, hypervascular, or poorly demarcated. The biological interpretation of UHFUS must therefore remain probabilistic and contextual. Sonography depicts macroscopic architecture, interfaces, and flow; histopathology evaluates cytology, growth pattern, perineural or lymphovascular invasion, and other microscopic determinants that cannot be resolved sonographically.

Within a multimodal diagnostic pathway, UHFUS provides high-resolution information on superficial lesion architecture and tissue–plane relationships, complementing clinical examination and histopathology, while CT and MRI remain necessary for deeper extension, bone involvement, nodal disease, and complex anatomical spread in future studies [47].

The dual-frequency acquisition strategy used in this series deserves consideration. The 70 MHz probe was used to maximize depiction of very superficial structures and fine anatomical interfaces, whereas the 48 MHz probe allowed greater depth of interrogation. Both probes were incorporated into the same examination, and the final qualitative interpretation integrated information obtained at both frequencies. Accordingly, the present study was neither designed nor powered to determine the incremental diagnostic value or superiority of either transducer. Their combined use should therefore be regarded as an acquisition strategy based on the expected physical trade-off between resolution and penetration, rather than as a formally demonstrated diagnostic advantage of dual-frequency imaging. Several methodological limitations should be acknowledged. The retrospective, single-center design and the use of one highly experienced operator limit external generalizability. The same radiologist performed the original examinations and the retrospective image review; although the review was conducted on anonymized examinations without access to the histopathological result or original ultrasound report, recall and reader-related bias cannot be completely excluded. The reviewer remained aware of the clinical indication, and the pathologist worked within the usual multidisciplinary clinical context rather than under blinded experimental conditions. No independent second-reader assessment, repeatability analysis, or dedicated histological re-review was performed. A further limitation is potential spectrum and selection bias. The final analytical cohort included only technically interpretable examinations of accessible lesions with histopathological confirmation. Consequently, the feasibility observed in the analyzed cohort should not be interpreted as evidence that UHFUS can be successfully performed in all patients presenting with oral cavity lesions. When calculated using all 137 attempted examinations as the denominator, technical feasibility was 96.4%, with five examinations excluded because of inadequate image or cine-loop quality. Patients with inaccessible lesions, inability to tolerate probe positioning, or absence of histopathological confirmation may further limit the generalizability of these findings. The study was deliberately qualitative and did not report feature frequencies, diagnostic performance metrics, or formal imaging–pathology agreement. Moreover, quantitative UHFUS measurements were not matched prospectively to histological sections along an identical predefined anatomical axis, and tissue shrinkage or deformation resulting from fixation and specimen processing was not specifically modeled. Therefore, the present data should not be interpreted as a quantitative validation of UHFUS measurements against histopathology, particularly for depth-related parameters. Furthermore, the study is subject to spectrum and selection bias. Eligibility favored clinically accessible lesions and patients able to tolerate intraoral probe positioning, while five of the 137 UHFUS examinations were excluded because image and/or cine-loop quality was insufficient for reliable retrospective assessment. Technical feasibility was therefore 96.4% (132/137) among attempted examinations and should not be extrapolated to an unselected population of patients with oral cavity lesions, particularly those with poorly accessible posterior lesions, severe gag reflex, limited mouth opening, pain, or other anatomical constraints. Five histologically confirmed illustrative cases were selected from the study cohort for educational representativeness and should not be interpreted as a statistically representative subsample. The gingival fistula was an additional clinically confirmed illustrative case outside the analytical cohort and was included solely to demonstrate the sonographic appearance of a superficially extending fistulous tract.

These limitations are balanced by several strengths: consecutive case identification within the study period, complete histopathological confirmation for the 132-lesion source cohort, standardized retrospective data extraction using predefined imaging variables, availability of both static images and cine loops, use of B-mode and Color Doppler in all cases, and joint radiological–pathological interpretation of the qualitative patterns. Future work should retain this structured layer-based framework while introducing prospective enrollment, dedicated intraoral probes, predefined acquisition settings, independent blinded readers, quantitative vascular and gray-scale biomarkers, and reproducibility testing. Studies should also distinguish clearly between lesion thickness, tumor thickness, and depth of invasion, and should evaluate whether the added information changes the biopsy strategy, surgical planning, margin status, or follow-up decisions.

In conclusion, this study supports UHFUS as a complementary method for the qualitative characterization of selected oral cavity lesions. Across the reviewed cohort, the technique provided information on lesion morphology, internal structure, vascular pattern, plane of origin, and relationship with adjacent superficial anatomy. The illustrative cases show that these findings can refine clinical understanding of reactive, benign, and malignant processes, while also demonstrating that no single sonographic feature is diagnostic in isolation. The most appropriate clinical role is therefore as a high-resolution component of a multimodal pathway that integrates clinical examination, pathology, and deeper cross-sectional imaging when required.

## 5. Conclusions

Intraoral UHFUS provides a high-resolution, layer-based method for evaluating selected oral cavity lesions. Its fundamental strength derives from matching the physics of ultra-high frequency imaging to the anatomy of superficial oral disease. By prioritizing spatial resolution over penetration, UHFUS can depict mucosa, submucosa, muscular planes, glandular structures, periodontal interfaces, and cortical bone boundaries in accessible oral subsites.

In this single-center observational study with histopathological correlation, UHFUS allowed structured characterization of benign and malignant lesions according to site, morphology, margins, echogenicity, echostructure, Doppler vascularity, and layer involvement. Among benign/reactive lesions, observed sonographic features included regular morphology, well-defined margins, and preservation of deeper planes, whereas malignant lesions showed a spectrum of findings that included irregular margins, heterogeneous echostructure, hypoechoic components, increased vascularity, and mucosal or submucosal involvement. These descriptive observations were not based on formal frequency comparisons and should not be interpreted as statistically validated criteria for differentiating benign from malignant lesions. However, histopathology remains essential, because sonographic architecture cannot fully define biological behavior.

The main contribution of the study is the application of a structured, layer-based assessment framework across a heterogeneous spectrum of oral cavity lesions. UHFUS should be regarded as a complementary imaging tool rather than a replacement for biopsy, histopathology, CT, or MRI. Potential areas for future clinical evaluation include pre-biopsy characterization, assessment of superficial lesion extent, depth-related assessment, procedural planning, and follow-up of selected oral lesions; however, the clinical impact of these applications was not evaluated in the present study and requires prospective validation. The technique remains limited by operator dependence, reduced penetration, technical difficulty in posterior and lateral regions, patient tolerance, and the need for standardized acquisition and reporting. Prospective studies incorporating quantitative histopathological correlation, independent readers, reproducibility assessment, and clinically relevant management endpoints are required before these potential applications can be translated into established clinical indications.

## Figures and Tables

**Table 1:** Demographic, anatomical, and histopathological characteristics of the 132-patient study cohort. Age is reported as mean ± standard deviation; categorical variables are reported as absolute numbers and percentages.

Characteristic	Value
Patients/lesions	132/132
Age, years	65.2 ± 12.7
Female	47 (35.6%)
Male	85 (64.4%)
Benign/reactive	57 (43.2%)
Malignant	75 (56.8%)
Tongue	35 (24.2%)
Buccal mucosa	32 (24.2%)
Gingiva	23 (17.4%)
Lip	7 (5.3%)
Hard palate	8 (6.1%)
Sublingual region	15 (11.4%)
Other oral cavity sites	12 (9.1%)

## Data Availability

The data presented in this study are available on request from the corresponding author.
